# Characteristics of submucosal leiomyomas that could cause severe hemorrhage with relugolix: an observational study

**DOI:** 10.1186/s12905-023-02241-2

**Published:** 2023-03-15

**Authors:** Yoshimitsu Wada, Yuji Takei, Takumi Minezumi, Hiroto Hirashima, Hiroyuki Fujiwara

**Affiliations:** 1 Department of Obstetrics and Gynecology, Sano Kosei General Hospital, 1728 Horigome, Sano, Tochigi, 327-8511 Japan; 2 Department of Obstetrics and Gynecology, Jichi Medical University, 3311-1 Yakushiji, Shimotsuke, Tochigi, 329-0498 Japan

**Keywords:** Adverse events, Gonadotropin-releasing hormone receptor antagonist, Relugolix, Uterine hemorrhage, Uterine leiomyoma, Submucosal leiomyoma

## Abstract

**Background:**

Relugolix, an oral gonadotrophin-releasing hormone receptor antagonist, was launched in Japan in 2019. Although there have been several studies on relugolix for leiomyomas, few have focused on submucosal leiomyomas. Submucosal leiomyomas cause bleeding more frequently than leiomyomas in other locations. There is only one case report described a patient treated for a submucosal leiomyoma with relugolix who developed severe hemorrhage. However, it remains unclear which characteristics of submucosal leiomyomas can lead to severe hemorrhage. Thus, the aim of this study was to investigate the characteristics of submucosal leiomyomas that would cause severe hemorrhage when treated with relugolix.

**Methods:**

We retrospectively reviewed records of patients who underwent treatment for submucosal leiomyoma with relugolix (40 mg once daily for up to 6 months) in our institute between December 2019 and September 2021. We evaluated the clinical course and characteristics of submucosal leiomyoma in patients who developed severe hemorrhage.

**Results:**

A total of 17 patients were treated for submucosal leiomyoma with relugolix. Two patients developed severe hemorrhage and required emergent surgery and blood transfusions. Only those two of the 17 patients had a submucosal leiomyoma of the International Federation of Gynecology and Obstetrics (FIGO) type 0, which has a stalk. In the remaining 15 patients who had FIGO type 1 or 2 leiomyoma, hemorrhage did not occur.

**Conclusions:**

Our study suggests that the use of relugolix for FIGO type 0 leiomyomas may be associated with a risk of hemorrhage. However, relugolix may be a safe and effective treatment option for patients with FIGO type 1 or 2 leiomyomas.

## Introduction

Uterine leiomyomas are common benign pelvic tumors in females. The age-standardized incidence rate is 9.2 per 1000 woman-years [1]. Uterine leiomyomas can cause abnormal bleeding, menorrhagia, pain, and infertility [2, 3]. Submucosal leiomyomas lead to anemia more frequently than leiomyomas in other locations [4]. Treatment options for uterine leiomyomas include medical therapy, uterine artery embolization, focused ultrasound surgery, and surgery (hysterectomy or myomectomy), depending on patient's symptoms, size and location types of leiomyomas, and fertility preservation.

Relugolix, an oral gonadotrophin-releasing hormone (GnRH) receptor antagonist for the treatment of uterine leiomyomas, was launched in Japan in 2019. Relugolix prevents the anterior lobe of the pituitary from secreting gonadotrophin, decreases estradiol and progesterone levels within days, and induces amenorrhea [2]. Unlike GnRH agonists, relugolix does not cause transient increases in gonadotropin, estradiol, and progesterone levels, known as flare-up. This lack of flare-up is considered a favorable feature of relugolix [2].

There have been five randomized controlled trials on relugolix for leiomyomas, including two that focused on the combination of relugolix, estradiol, and norethindrone acetate [2, 5–8]. Those studies reported that relugolix improved leiomyoma-related symptoms, such as menstrual bleeding, anemia, and pain, and reduced the size of leiomyomas. The majority of adverse events reported were mild to moderate [2, 5–8]. However, there is limited literature on the use of relugolix for submucosal leiomyomas. One case report described a patient treated for a submucosal leiomyoma with relugolix, who developed severe hemorrhage and prolapse of a pedunculated myoma through the uterine cervix during treatment [9]. The package insert for relugolix states that severe hemorrhage may occur when it is used for submucosal leiomyoma [10]. However, in our experience, severe hemorrhage did not occur in some cases even when relugolix was used for treatment of submucosal leiomyomas. It remains unclear which characteristics of submucosal leiomyomas can lead to severe hemorrhage.

The aim of this study was to investigate the characteristics of submucosal leiomyomas that would cause severe hemorrhage when treated with relugolix.

## Methods

We retrospectively reviewed the medical records of all patients who underwent treatment for submucosal leiomyoma with relugolix (40 mg once daily for up to 6 months) in Sano Kosei General Hospital (Tochigi, Japan), a local general hospital, between December 2019 and September 2021. Our study was approved by the ethics committee of Sano Kosei General Hospital [approval number: 202120 (Feb. 7th, 2022)].

**Fig. 1:**
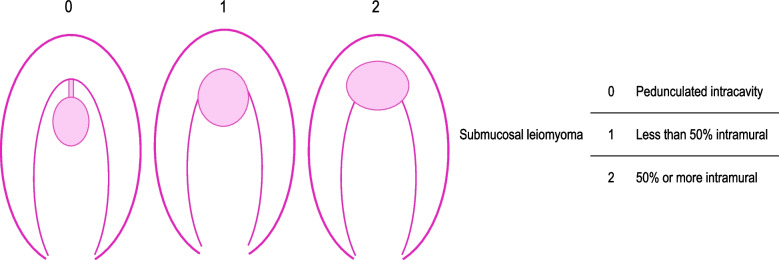
The International Federation of Gynecology and Obstetrics leiomyoma subclassification system of submucosal leiomyoma [11]

**Fig. 2:**
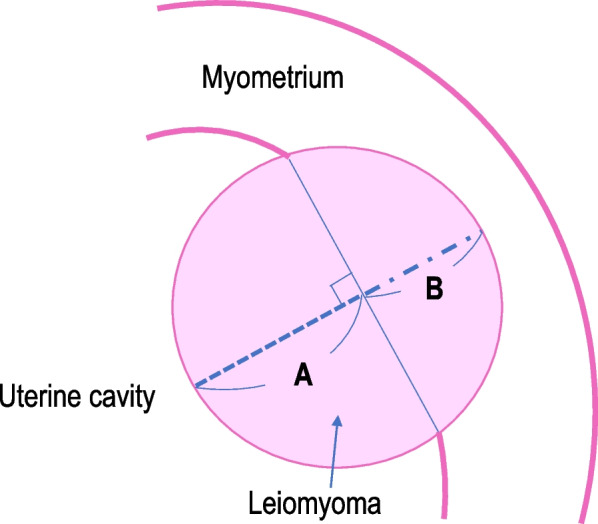
Protrusion rate of submucosal leiomyoma Cross-sectional image of the leiomyoma protruding most into the intrauterine cavity shown by ultrasound or MRI. **A**: the length of a leiomyoma protruding into the intrauterine cavity. **B**: the length of a leiomyoma confined to the myometrium. Protrusion rate = A / (A + B) × 100 (%).

We examined severe vaginal hemorrhage and other adverse events from medical records. Severe vaginal hemorrhage was defined as requiring blood transfusion or emergency surgery. We retrieved baseline characteristics of patients as follows: age, parity, body mass index (BMI), leiomyoma-related symptoms (menorrhagia and dysmenorrhea), hemoglobin (Hb), ultrasound and magnetic resonance imaging (MRI) findings of leiomyomas (diameter, volume, the International Federation of Gynecology and Obstetrics [FIGO] leiomyoma subclassification system type [Fig. 1] [11], and protrusion rate of submucosal leiomyomas), and treatment periods. In cases where patients had multiple leiomyomas, we evaluated the diameter, volume, FIGO subclassification, and protrusion rate of the largest submucosal leiomyoma. These parameters were measured using ultrasound or MRI. We preferably used MRI before ultrasound to calculate the protrusion rate and to decide FIGO subclassification. Leiomyoma volume was assessed only in patients who underwent MRI. The volume of leiomyoma was calculated by the formula: (the maximal longitudinal diameter × anteroposterior diameter × transverse diameter × π / 6), using T2-weighted MRI. The protrusion rate of leiomyoma was evaluated using a method previously described [12]. On a cross-sectional image of the leiomyoma protruding most into the intrauterine cavity, the length of the part protruding into the intrauterine cavity (A) and part confined to the myometrium (B) were measured (Fig. 2). The protrusion rate was calculated by: (A / (A + B) × 100).

We evaluated Hb, diameter of leiomyomas, and reduction rate after treatment. The reduction rate was calculated with the maximum diameter of the leiomyoma measured on ultrasound or MRI by the formula: ([pre-treatment diameter-post-treatment diameter] / pre-treatment diameter × 100). We evaluated the reduction rate of the largest submucosal leiomyoma, if a patient had multiple leiomyomas. The differences of Hb and diameter of leiomyomas between pre- and post-treatment were analyzed. We performed imaging studies using ultrasound (Voluson P8; General Electric Company, Boston, Massachusetts) with a 2.9–9.7-MHz endovaginal probe and a 2–5-MHz convex probe, and MRI (Vantage Galan 3T; CANON MEDICAL SYSTEMS CORPORATION, Tochigi, Japan).

We described the baseline characteristics of patients. Continuous variables are presented as the median with interquartile range (IQR), and categorical variables are presented as the number with proportion. The differences of Hb and diameter of leiomyomas between pre- and post-treatment were evaluated using a Wilcoxon Signed Rank Test. We conducted complete case analyses when variables included missing data. All statistical analyses were performed with the statistical package R 3.5.0. (The R Foundation, Vienna, Austria). A p-value of 0.05 or less was considered significant.

## Results

A total of 17 patients were enrolled. Table 1 shows the baseline characteristics of patients before treatment with relugolix. All patients had menorrhagia. Pre-treatment Hb (median [IQR]) was 10.2 (7.9–10.6) g/dL. All patients had anemia (Hb ≦ 12.0 g/dL) and received oral iron with relugolix. The median (IQR) diameter and volume of submucosal leiomyomas were 39.0 (28.0–54.0) mm and 30.1 (10.8–55.9) cm^3^, respectively. The protrusion rate of leiomyoma (only FIGO type 1 or 2) (median [IQR]) was 62 (49–69) %. The proportions of FIGO type 0, 1, and 2 were 2 (12%), 11 (65%), and 4 (24%), respectively.

**Table 1:** Baseline characteristics of patients before treatment with relugolix

	Total (*N* = 17)
Age (years), median (IQR)	46.0 (43.0–48.0)
Parity, median (IQR)	1.0 (0.0–2.0)
BMI (kg/m^2^), median (IQR)	22.0 (20.5–26.2)
Menorrhagia, n (%)	17 (100)
Dysmenorrhea, n (%)	4 (24)
Hemoglobin (g/dL), median (IQR)	10.2 (7.9–10.6)
Diameter of leiomyoma^a^ (mm), median (IQR)	39.0 (28.0–54.0)
Volume of leiomyoma^a,b^ (cm^3^), median (IQR)	30.1 (10.8–55.9)
Leiomyoma protrusion rate^a,c^ (%), median (IQR)	62.0 (49.0–69.0)
FIGO type of submucosal leiomyoma^a^, n (%)	
Type 0	2 (12)
Type 1	11 (65)
Type 2	4 (24)
Treatment periods (days), median (IQR)	126.0 (84.0–170.0)

*BMI*, Body mass index, *FIGO* The international federation of gynecology and obstetrics, *IQR* Interquartile range

^a^In cases where patients had multiple leiomyomas, we evaluated the largest submucosal leiomyoma

^b^There were missing variables in the volume of leiomyoma (three patients) because they did not undergo magnetic resonance imaging

^c^Protrusion rate of submucosal leiomyoma of FIGO type 1 or 2

Table 2 shows the outcomes of patients after treatment with relugolix. There were two (12%) patients who developed severe hemorrhage and subsequently received emergent vaginal myomectomy and transfusions. There was no other major adverse event in this study. All 15 patients with FIGO type 1 or 2 showed improvement of menorrhagia. Post-treatment Hb was significantly elevated, compared with pre-treatment (*P* = 0.003). The post-treatment diameter of leiomyomas was significantly decreased compared with pre-treatment (*P* < 0.001). The median (IQR) reduction rate was 25.0 (8.1–29.6) %. In 15 patients with FIGO type 1 or 2 (excluding type 0), the median (IQR) reduction rate was 25.7 (12.4–30.8) %.

**Table 2:** Outcomes of patients after treatment with relugolix

	Total (*N* = 17)
Severe vaginal bleeding^a^, n (%)	2 (12)
Hemoglobin (g/dL), median (IQR)^b^	13.1 (12.1–14.1)
Diameter of leiomyoma^c^ (mm), median (IQR)	26.0 (23.0–47.0)
Reduction rate of leiomyoma^c^ (%), median (IQR)	25.0 (8.1–29.6)

*IQR* Interquartile range

^a^The two patients developed severe hemorrhage and subsequently received emergent vaginal myomectomy and transfusions

^b^There were missing variables in post-treatment hemoglobin (two patients)

^c^In cases where patients had multiple leiomyomas, we evaluated the largest submucosal leiomyoma

**Fig. 3:**
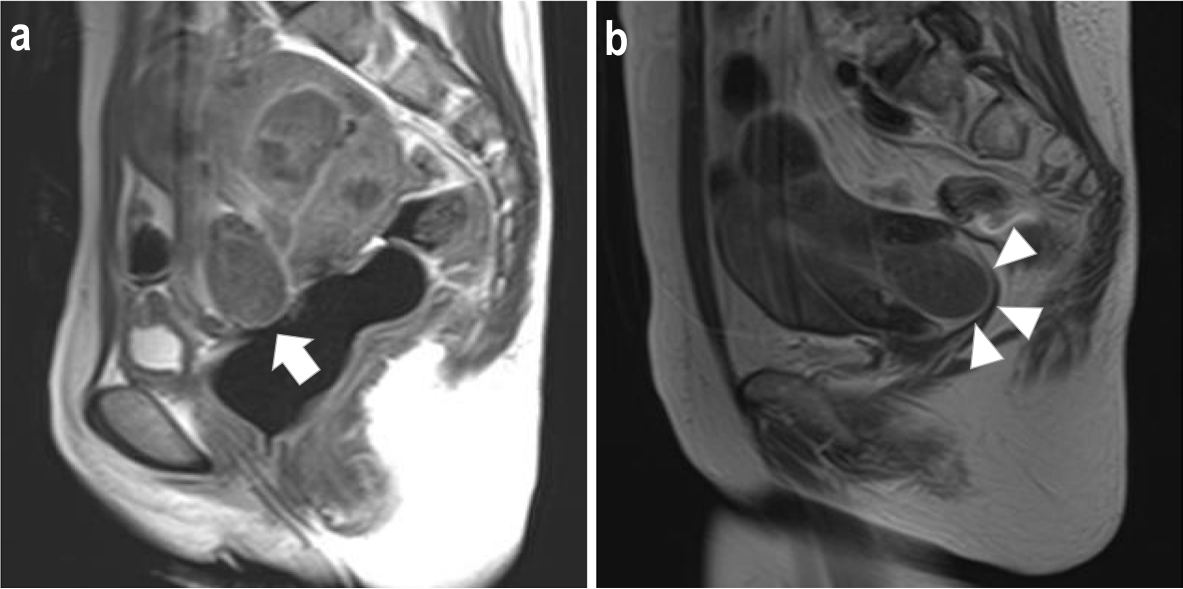
The two patients who developed severe hemorrhage **a**. Patient 1 (post-treatment) T2-weighted MRI sagittal view. Arrow: The pedunculated myoma prolapsing through the cervix (33 × 27 × 24 mm) **b**. Patient 2 (pre-treatment) T2-weighted MRI sagittal view. Arrowhead: The pedunculated myoma prolapsing through the cervix (61 × 43 × 31 mm).

The two patients developed severe hemorrhage, and received emergent vaginal myomectomy and blood transfusions (Table 3). One of the two patients (patient 1) did not undergo MRI before relugolix started. We performed only ultrasound for this patient, and considered that she had multiple intramural leiomyomas without submucosal leiomyoma. On day 27 of treatment, she developed severe hemorrhage, and pelvic examination and MRI revealed a pedunculated myoma prolapsing through the cervix that was 33 × 27 × 24 mm (Fig. 3a). MRI showed that the submucosal leiomyoma had a stalk. A blood test showed Hb of 7.8 g/dL and she underwent vaginal myomectomy on the same day. In the other patient (patient 2), she had a pedunculated myoma prolapsing through the cervix that was 61 × 43 × 31 mm before relugolix started (Fig. 3b). She received relugolix to prevent menstrual bleeding before the scheduled surgery. However, on day five of treatment, she developed severe hemorrhage, and a blood test showed Hb of 7.3 g/dL. Pelvic examination revealed bleeding originating from the surface of the prolapsing leiomyoma. She also underwent vaginal myomectomy on the same day. The postoperative courses of the two patients were favorable. In our study, only two patients developed severe hemorrhage, and underwent emergent surgery and transfusion. They had a submucosal leiomyoma of FIGO type 0. On the other hand, in the remaining 15 patients had a submucosal leiomyoma of FIGO type 1 or 2, hemorrhage did not occur.

**Table 3:** The two patients who developed severe hemorrhage on relugolix

	Age (years)	Parity	BMI (kg/m^2^)	Hb (g/dL) (pre-treatment)	Hb (g/dL) (post-treatment)	Leiomyoma diameter (mm) (pre-treatment)	Leiomyoma diameter (mm) (post-treatment)	FIGO type of submucosal leiomyoma	Hemorrhage occurrence from start of relugolix (days)	Intervention
Patient 1	46	1	18.0	9.9	7.8	42	33	0	27	Transfusions, Emergent vaginal myomectomy
Patient 2	50	2	26.2	10.4	7.3	61	61	0	5	Transfusions, Emergent vaginal myomectomy

*BMI* Body mass index, *FIGO* The international federation of gynecology and obstetrics, *Hb* Hemoglobin

## Discussion

In our study, severe adverse events occurred in two patients who developed hemorrhage, and required emergent surgery and transfusions. Only those two patients had a submucosal leiomyoma of FIGO type 0. On the other hand, hemorrhage did not occur in the remaining 15 patients who had a submucosal leiomyoma of FIGO type 1 or 2.

Randomized controlled trials showed that relugolix decreased menstrual blood loss and increased the Hb level in patients with leiomyomas [2, 5–8]. In those studies, almost all adverse events were mild to moderate. A recent retrospective study also did not show severe adverse events in patients who received relugolix for leiomyomas [13]. However, in our study, two patients developed severe hemorrhage. In previous studies, only one patient treated for a leiomyoma with relugolix and six patients treated with a GnRH agonist showed prolapse of a pedunculated myoma through the cervix and bleeding during treatment [9, 14–17]. All of the seven patients had submucosal leiomyomas and one case was FIGO type 0 [9], but other patients had an unknown FIGO type [14–17]. In our cases, only the two patients had a submucosal leiomyoma of FIGO type 0. Relugolix use for submucosal leiomyoma of FIGO type 0 may be associated with risks of hemorrhage and prolapse. On the other hand, relugolix for patients with submucosal leiomyoma of FIGO type 1 or 2 may be safe. Our study demonstrated that in the 15 patients with FIGO type 1 or 2 leiomyomas, all showed improvements in menorrhagia and the median reduction rate was 25.7%. These findings suggest that relugolix can be an effective treatment option for patients with FIGO type 1 or 2 leiomyomas.

Our patient 1 developed hemorrhage and prolapse of a pedunculated myoma through the cervix on treatment day 27 with the shrinkage of leiomyoma. In a previous study, the mechanism of leiomyoma prolapse was considered to involve rapid decreases in the leiomyoma volume [9]. Patient 1 did not undergo MRI before relugolix started, and we considered that she had multiple intramural leiomyomas without submucosal leiomyoma based on the findings of ultrasound. A previous study reported that MRI was useful for the evaluation of submucosal leiomyoma [18]. MRI should have been conducted before using relugolix to rule out a submucosal leiomyoma of FIGO type 0 if a patient has multiple leiomyomas or ultrasound examination shows unclear images. Patient 2 had a pedunculated myoma prolapsing through the cervix before relugolix started and developed hemorrhage on treatment day five. The cause of hemorrhage in patient 2 was considered to be another mechanism. Relugolix reduces the estradiol level within three days [6]. This decrease may make the leiomyoma and its stalk fragile and more prone to bleeding. Thus, patient 2 may have developed hemorrhage because the fragile surface of the leiomyoma got in contact with the vaginal wall. These two patients could have developed severe hemorrhage without using relugolix. However, we consider that relugolix increases the possibility of prolapsing and bleeding of submucosal leiomyomas of FIGO type 0 for the above reasons.

This study has a few limitations, as follows. First, this was a retrospective study conducted in a single institute, and included a relatively small sample size. However, to the best of our knowledge, there has been no study limited to submucosal leiomyoma treated with relugolix other than one case report [9], and our study is the first to describe multiple patients. Second, we could not evaluate mild to moderate adverse events, such as menopausal symptoms, because data were missing for some patients. Third, the evaluation of FIGO subclassification and protrusion rate in two patients who did not undergo MRI was limited to ultrasound imaging. This may have had an impact on the accuracy of FIGO subclassification and protrusion rate evaluations.

## Conclusions

In conclusion, our study showed two patients with prolapse of a pedunculated myoma through the uterine cervix, who developed severe hemorrhage, and required emergent surgery and transfusions after relugolix started. Only those two patients had a submucosal leiomyoma of FIGO type 0 among all 17 cases. We cannot recommend the use of relugolix for patients with FIGO type 0 submucosal leiomyomas because of the risk of hemorrhage. However, relugolix may be a safe and effective treatment option for patients with FIGO type 1 or 2 submucosal leiomyomas.

## Data Availability

The datasets used and analyzed during the current study are available from the corresponding author on reasonable request.

## Abbreviations

GnRH: Gonadotropin-releasing hormone
BMI: Body mass index
MRI: Magnetic resonance imaging
FIGO: The International Federation of Gynecology and Obstetrics
Hb: Hemoglobin; IQR: interquartile range
